# Detection of quantitative trait loci controlling grain zinc concentration using Australian wild rice, *Oryza meridionalis*, a potential genetic resource for biofortification of rice

**DOI:** 10.1371/journal.pone.0187224

**Published:** 2017-10-27

**Authors:** Ryo Ishikawa, Masahide Iwata, Kenta Taniko, Gotaro Monden, Naoya Miyazaki, Chhourn Orn, Yuki Tsujimura, Shusaku Yoshida, Jian Feng Ma, Takashige Ishii

**Affiliations:** 1 Graduate School of Agricultural Science, Kobe University, Kobe, Japan; 2 Institute of Plant Science and Resources, Okayama University, Kurashiki, Japan; University of Warwick, UNITED KINGDOM

## Abstract

Zinc (Zn) is one of the essential mineral elements for both plants and humans. Zn deficiency in human is one of the major causes of hidden hunger, a serious health problem observed in many developing countries. Therefore, increasing Zn concentration in edible part is an important issue for improving human Zn nutrition. Here, we found that an Australian wild rice *O*. *meridionalis* showed higher grain Zn concentrations compared with cultivated and other wild rice species. The quantitative trait loci (QTL) analysis was then performed to identify the genomic regions controlling grain Zn levels using backcross recombinant inbred lines derived from *O*. *sativa* ‘Nipponbare’ and *O*. *meridionalis* W1627. Four QTLs responsible for high grain Zn were detected on chromosomes 2, 9, and 10. The QTL on the chromosome 9 (named *qGZn9*), which showed the largest effect on grain Zn concentration was confirmed with the introgression line, which had a W1627 chromosomal segment covering the *qGZn9* region in the genetic background of *O*. *sativa* ‘Nipponbare’. Fine mapping of this QTL resulted in identification of two tightly linked loci, *qGZn9a* and *qGZn9b*. The candidate regions of *qGZn9a* and *qGZn9b* were estimated to be 190 and 950 kb, respectively. Furthermore, we also found that plants having a wild chromosomal segment covering *qGZn9a*, but not *qGZn9b*, is associated with fertility reduction. *qGZn9b*, therefore, provides a valuable allele for breeding rice with high Zn in the grains.

## Introduction

Zinc (Zn) as an essential micronutrient for all living organisms. Zn is involved in many biochemical functions of proteins, lipids, and nucleic acids [1]. An insufficient level of Zn in human body causes multiple clinical manifestations (i.e., impaired immune function, growth retardation, hair loss, eye and skin lesions, and loss of appetite) [2]. Thus, Zn deficiency is now recognized as one of the causes of hidden hunger, that is, malnutrition caused by vitamin and mineral deficiencies [3]. To mitigate hidden hunger, biofortification of grain Zn (increasing grain Zn levels based on genetic approaches) in many crops, such as rice, wheat, and maize, has been attempted [2, 4–6]. In addition, applications of fertiliser have been tested [7–9]. According to the recent study on field experiments of wheat, Zn application and field management of nitrogen and phosphorus are important for increasing grain Zn level together with Zn-responsive genotypes [9]. These challenges to increase grain Zn level in crops by efficient use of fertiliser and breeding are important for future agriculture.

Rice is a staple food that supports more than half of the world’s population [10]. People in many developing Asian countries with high rice consumption have low levels of Zn, and the effects of Zn deficiency are most severe in pregnant women and children. To meet daily Zn requirements, a diet consisting of Zn-rich foods and preventive supplementation can compensate for Zn deficiency, but these approaches are impractical in many developing countries. Therefore, breeding of new rice cultivars with enhanced levels of grain Zn is one of the most cost effective and sustainable strategies for preventing hidden hunger.

Several studies aimed at controlling grain Zn concentration in rice used transgenic approaches. Constitutive overexpression of the nicotianamine synthase gene family, *OsNAS1 or OsNAS2*, in rice has been shown to increase grain iron and Zn concentrations [11]. A following study showed that *OsNAS2* overexpression can be used for biofortified rice based on the evaluation of agro-morphological traits of cultivars with the transgene [12]. Identification of the genes involved in Zn translocation or homeostasis has also provided opportunities to test their application in biofortified rice [13–16]. For example, *OsHMA2*, encoding rice (*O*. *sativa*) heavy metal ATPase2, showed high expression in the roots and nodes at the vegetative and reproductive stages, respectively [15]. Similarly, *OsZIP3*, encoding a member of ZIP (ZRT, IRT-like protein), was found to be expressed in the node [16]. These two transporters play independent roles on preferential distribution of Zn to the developing tissues in nodes [15,16]. The functional analyses of the Zn transporters suggest the possibility of manipulation of Zn translocation and distribution, although cultivation of genetically modified plants with these genes still requires deregulation efforts in many countries [2].

Alternative approaches for enhancing grain Zn levels employ the germplasm of natural genetic variations within the genus *Oryza* [2,17]. Due to large genetic variations and growing environments, the wild relatives of rice species are suggested to have a great potential for conferring better agricultural traits such as resistance to abiotic and biotic stresses for future breeding [18–20]. In the genus *Oryza*, five wild (*O*. *rufipogon*, *O*. *glumaepatula*, *O*. *meridionalis*, *O*, *barthii*, and *O*. *longistaminata*) and two cultivated (*O*. *sativa* and *O*. *glaberrima*) rice species are classified as A-genome species [21,22]. Although there are some reproductive barriers between them, recurrent backcrossing to cultivated rice can be used for conventional breeding. Thus, rice relatives with high grain Zn concentrations may be useful in breeding biofortified rice.

In this study, we found that an Australian wild rice *O*. *meridionalis* had a higher grain Zn concentration. We generated backcross recombinant inbred lines (BRILs) between *O*. *sativa* ‘Nipponbare’ and *O*. *meridionalis* acc. W1627. Four quantitative trait locus (QTL) responsible for high grain Zn concentration were detected. Further genetic analysis on *qGZn9*, one of the four QTLs with the largest phenotypic effect, was carried out to specify the major useful genes for Zn biofortification in rice.

## Materials and methods

### Plant materials

Two rice cultivars, *O*. *sativa* Japonica ‘Nipponbare’ and Indica ‘IR36’ and one accession each from A-genome wild rice species, *O*. *rufipogon* W630, *O*. *glumaepatula* W1169, *O*. *meridionalis* W1627, and *O*. *barthii* W1152, were used for the initial evaluation of grain Zn concentration (S1 Table). In addition to W1627, we used 10 accessions of *O*. *meridionalis* for the measurement of grain Zn concentration (S1 Table). These wild and cultivated plants were grown in 3.5-L pots under greenhouse conditions with natural light. Three months after germination, a short-day treatment was applied to the wild rice accessions to induce flowering.

To generate BRILs, we first crossed *O*. *sativa* ‘Nipponbare’ with *O*. *meridionalis* W1627, and from their single F_1_ plant, 14 BC_1_F_1_ plants were obtained. They were further backcrossed with ‘Nipponbare’, and a total of 210 BC_2_F_1_ plants (15 plants each from the 14 BC_1_F_1_ plants) were produced. From these, BRILs at the BC_2_F_7_ generation were developed by the single seed descent method. The plants were not artificially selected during the selfing process, but 59 lines were eliminated because of extremely late flowering, weak growth, or high sterility. As a result, a total of 151 BRILs were obtained. These lines were grown in the paddy field at Kobe University, and their grain Zn concentration was determined as described below.

### Determination of grain Zn concentration

The grains were de-hulled and dried at 70°C for 3 days. The samples were digested with concentrated nitric acid (60% [w/v]) at up to 140°C as described previously [23]. After dilution, Zn concentration was determined by inductively coupled plasma-mass spectrometry (ICP-MS 7700X, Agilent Technologies).

### QTL analysis for grain Zn concentration

The genomic DNA of 151 BRILs was extracted, and 164 polymorphic simple sequence repeat marker loci covering the whole rice genome were surveyed. The amplified fragments were electrophoresed in 4.0% polyacrylamide gels, and banding patterns were visualised by the silver staining method [24]. Interval mapping analysis was applied to detect putative QTLs with an LOD threshold value of 3.0 using WinQTL cartographer ver. 2.5 [25].

### Mapping analysis

A single BRIL of MN91 with the strongest W1627 allele increasing grain Zn concentration was further used (S1 Fig). This line was backcrossed with ‘Nipponbare’ and the plants only having heterozygous chromosomal constitutions at the target QTL region were selected to produce a fine mapping population. Plants used for the mapping experiments were grown in 3.5-L pots under greenhouse conditions together with control lines, ‘Nipponbare’, MN91, and W1627.

### Candidate gene analysis

The presence or absence of the candidate genes was examined based on the sequence reads of an introgression line having a W1627 chromosomal segment at the QTL. DNA was extracted from 100 mg fresh leaves using the DNeasy Plant mini kit (Qiagen), and 3 μg of the DNA was used for preparation of library for Illumina sequencing according to the protocol for the TruSeq Nano DNA LT Library Prep kit (Illumina). Sequence reads were obtained using HiSeq 2500 (Illumina). Using the Galaxy software (https://usegalaxy.org/), the short reads were aligned to the reference genome of the ‘Nipponbare’ cultivar from Rice Annotation Project Database (RAP-DB, http://rapdb.dna.affrc.go.jp/) [26].

### Evaluation of grain traits and fertility

The grain sizes (length, width, and thickness) and weight of each plant were measured based on the average values of 15 seeds. Seed production parameters (number of panicles and spikelets per panicle) were also measured. The spikelet number per panicle was estimated using the average values of five panicles. The fertility of plants in the progeny test was evaluated. The number of fertile seeds was counted against the total number of spikelets in a single panicle. The average ratio of two panicles was used as the ratio of fertility.

## Results

### *O*. *meridionalis* showed higher grain Zn concentration in A-genome rice species

**Fig 1:**
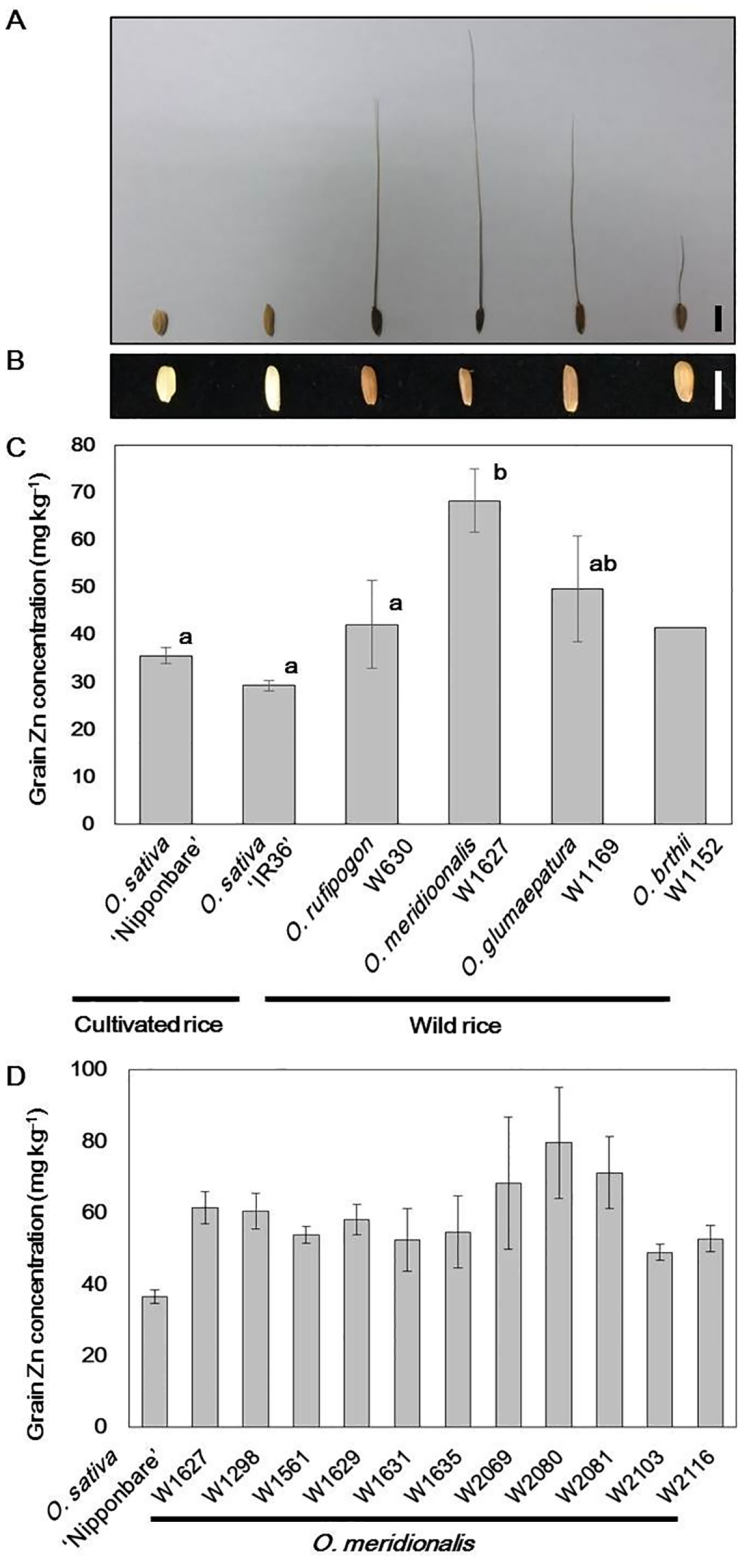
Grain Zn concentrations of cultivars and wild accessions in A-genome rice species. (A) Seeds and (B) grains of rice cultivars of *Oryza sativa* ‘Nipponbare’ and ‘IR36’ and four wild rice accessions: *O*. *rufipogon* W630, *O*. *meridionalis* W1627, *O*. *glumaepatula* W1169, and *O*. *barthii* W1152. Scale bars = 5 mm. (C) Grain Zn concentrations of two cultivars and four wild accessions. Data are presented as mean ± s.d. (n = 3). Mean values labelled with different letters are significantly different (Tukey’s test, *P* < 0.01). The average grain Zn concentration of two plants is shown for W1152. (D) Grain Zn concentration of 11 *O*. *meridionalis* accessions compared to *O*. *sativa* ‘Nipponbare’. Data are presented as mean ± s.d. (n = 3).

We compared Zn concentrations in brown rice (dehusked grain) among two cultivars of *O*. *sativa* Japonica ‘Nipponbare’ and Indica ‘IR36’ and one accession each from the A-genome wild rice species, *O*. *rufipogon*, *O*. *meridionalis*, *O*. *glumaepatula*, and *O*. *barthii* (S1 Table). The grain Zn concentrations of the two cultivated rice species were approximately 30–35 mg kg^-1^ (Fig 1C).

We found that the Australian wild accession *O*. *meridionalis* W1627 had the highest grain Zn concentration (68.3 ± 6.7 mg kg^-1^) among the wild and cultivated rice accessions examined. To investigate whether the high grain Zn concentration is a species-specific phenomenon, we further examined grain Zn concentrations in additional 10 accessions of *O*. *meridionalis* collected in Australia (S1 Table). All 11 accessions of *O*. *meridionalis* showed higher concentrations than those of *O*. *sativa* ‘Nipponbare’ (Fig 1D), suggesting that high grain Zn concentration is a common trait of *O*. *meridionalis* accessions.

### Genome coverage of backcross recombinant inbred lines and determination of their grain Zn concentration

**Fig 2:**
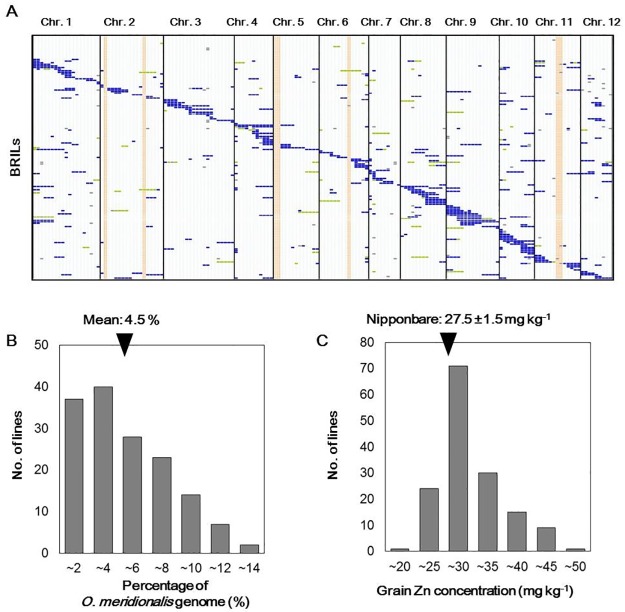
Genome coverage of backcross recombinant inbred lines (BRILs) and their grain Zn concentrations. (A) Graphical genotypes of 151 BRILs at BC_2_F_7_ between *O*. *meridionalis* W1627 and *O*. *sativa* ‘Nipponbare’ based on the 164 SSR loci. The chromosomal segments are indicated by colour: ‘Nipponbare’ homozygous (white), heterozygous (green), W1627 homozygous (blue), and unknown regions (grey). Chromosomal gaps not covered with W1627 homozygous segments of BRILs are shown in salmon. (B) Frequency distribution of the percentage of the *O*. *meridionalis* W1627 genomes found in the BRILs. (C) Frequency distribution of grain Zn concentration in the BRILs. The average grain Zn concentration of *O*. *sativa* ‘Nipponbare’ is shown.

To understand the genetic basis of the high grain Zn concentration of *O*. *meridionalis*, we produced a segregating population of BRILs between *O*. *sativa* ‘Nipponbare’ and *O*. *meridionalis* W1627 (Fig 2).

The chromosomal constitutions of 151 BRILs were analysed using 164 SSR markers covering the whole rice genome. These lines have several chromosomal segments from W1627 in the genetic background of *O*. *sativa* ‘Nipponbare’ (Fig 2A). The frequency distribution of the percentage of wild chromosomal segments introgressed in ‘Nipponbare’ is shown in Fig 2B. The average W1627 chromosomal segments were approximately 4.5% in the BRILs. We grew the BRILs in a paddy field, and their grain Zn concentrations were determined (Fig 2C). Grain Zn concentrations for *O*. *meridionalis* grown in the paddy field were not obtained, as it does not flower under natural day-length conditions in Japan. The average grain Zn concentration of ‘Nipponbare’ was 27.5 ± 1.5 mg kg^-1^. Most of the lines showed similar grain Zn levels to that of ‘Nipponbare’, and several lines showed high grain Zn concentrations such as *O*. *meridionalis*. A wide distribution of grain Zn concentrations was observed, suggesting that the difference in grain Zn concentration between ‘Nipponbare’ and W1627 is controlled quantitatively.

### Detection of QTLs for grain Zn concentration in *O*. *sativa* ‘Nipponbare’ and *O*. *meridionalis* W1627

To detect the genomic regions controlling grain Zn concentration, we carried out QTL analysis. Using genotype data from 164 SSR marker loci and grain Zn concentrations of each BRIL, four QTLs were detected; one each on chromosomes 9 (*qGZn9*) and 10 (*qGZn10*), and two on chromosome 2 (*qGZn2-1* and *qGZn2-2*) (Table 1, S1 Fig).

**Table 1:** Characteristics of the QTLs for grain Zn concentration detected in cultivated rice *Oryza sativa* ‘Nipponbare’ and Australian wild rice *O*. *meridionalis* W1627.

QTL	Chr.	QTL region	Source ^1)^	LOD	PV^2)^ (%)	Additive effect (mg kg^-1^)
*qGZn9*	9	RM24085-RM566	W1627	10.3	21.9	4.6
*qGZn10*	10	RM171-RM590	W1627	8.4	15.0	3.3
*qGZn2-1*	2	RM573	W1627	6.9	15.2	5.8
*qGZn2-2*	2	RM6	W1627	6.3	17.6	5.1

^1)^ Allele source increasing grain Zn concentration.

^2)^ Percentage of variance explained by the QTL.

At all four QTLs, W1627 alleles were found to increase grain Zn concentrations. Of these, *qGZn9* on chromosome 9 showed the highest effect explaining 21.9% of the total phenotypic variance with an LOD score of 10.3. These results confirm that the high grain Zn concentration of *O*. *meridionalis* W1627 involves several genes.

### Genetic dissection of the *qGZn9* candidate region

**Fig 3:**
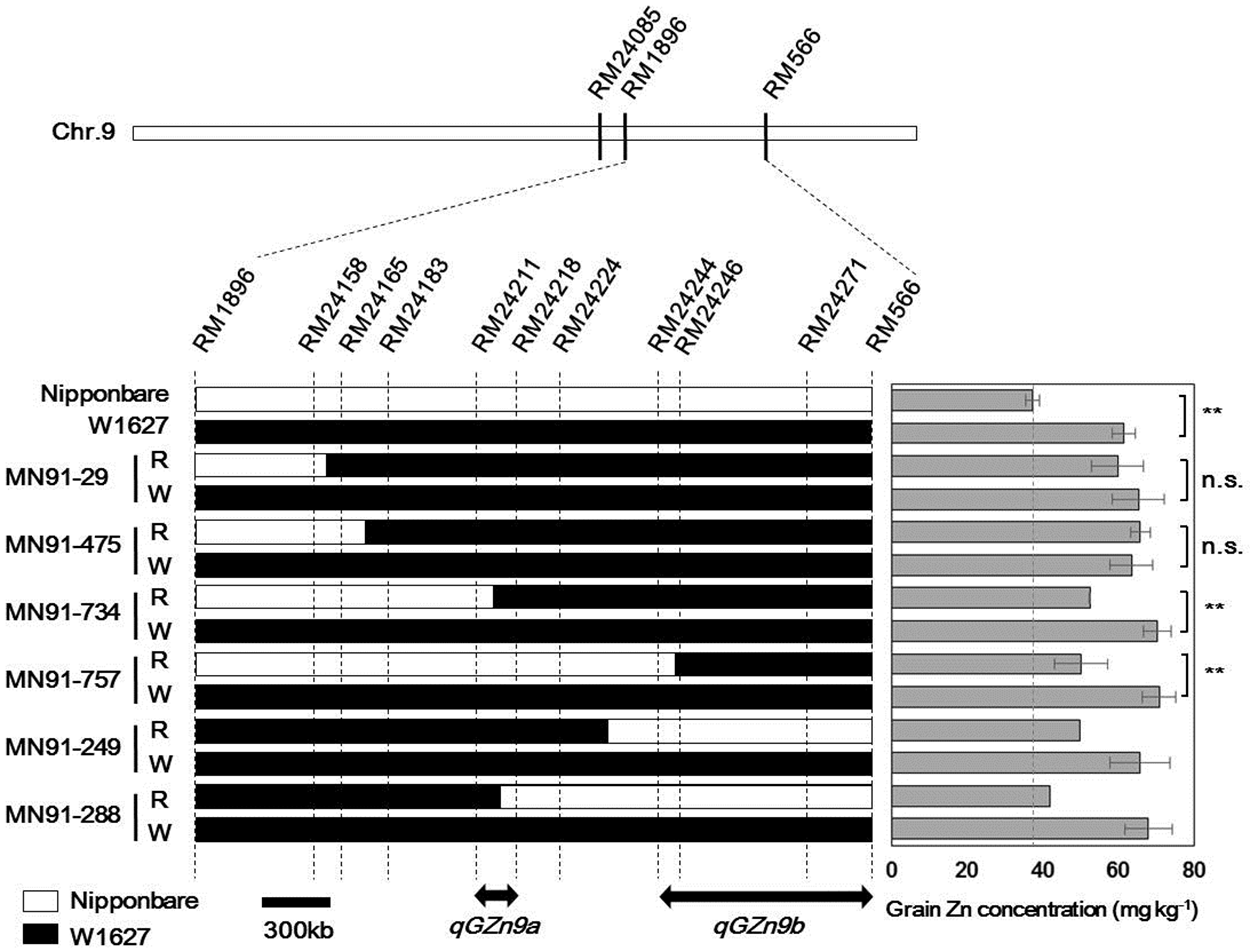
Two independent loci, *qGZn9a* and *qGZn9b*, identified in the putative *qGZn9* genomic region on chromosome 9. Graphical genotypes of two homozygous lines with wild (W) and recombinant (R) chromosomes, derived from six critical recombinants. White and black bars indicate ‘Nipponbare’ and W1627 chromosomal segments, respectively. Data are mean ± s.d. (n = 4). The average grain Zn concentrations of two plants from two recombinant lines nos. 249-R and 288-R are shown. n.s. and ** indicate not significant and significant at the 1% level by unpaired Student’s *t*-test, respectively.

To confirm the effect of *qGZn9* on grain Zn concentration, we chose a BRIL of MN91 that carries W1627 chromosomal segments on chromosome 1 and 9, covering the *qGZn9* region (S1 Fig). The grain Zn concentration of MN91 was 57.0 ± 10.2 mg kg^-1^, which is almost 1.5 times higher than that of *O*. *sativa* ‘Nipponbare’ (38.9 ± 2.7 mg kg^-1^). We confirmed that grain size is not likely to be the main cause of high grain Zn concentration controlled by *qGZn9*, as the grain size of MN91 was slightly smaller than that of ‘Nipponbare’ but much larger than that of W1627 (S2 Table). In addition, we found that parameters of seed production of MN91 were slightly higher than ‘Nipponbare’ (S3 Table), suggesting that the high grain Zn concentration of MN91 is not likely related to the smaller number of panicles or spikelets. However, a large difference in fertility was observed, suggesting that Zn might be accumulated due to the reduction in fertile seeds. Since the MN91 line has W1627 chromosomal segments in the genetic background of ‘Nipponbare’, it was further backcrossed with ‘Nipponbare’, and an introgression line (IL) having only a W1627 chromosomal segment covering *qGZn9* on chromosome 9 was produced. The IL showed a similar heading date to that of ‘Nipponbare’ (approximately 110 days after germination), but it exhibited a high grain Zn concentration of 71.2 ± 6.6 mg kg^-1^, confirming that the W1627 allele at *qGZn9* had a positive effect on grain Zn concentration. Next, we used two SSR markers flanking the *qGZn9* locus, RM24085 and RM566, for fine mapping. A total of 824 F_2_ plants were screened to identify recombinants having W1627 homozygous genotype at either of the two markers. Among 79 plants having recombination between the two markers, six critical plants (MN91, Nos. 29, 249, 288, 475, 734, and 757) were further self-pollinated to produce two types of homozygous plants with wild recombinant chromosomes (Fig 3).

Fig 3 shows the comparison of grain Zn concentration between two homozygous lines from the critical recombinants. All the six wild homozygous lines and W1627 showed much higher grain Zn concentrations (> 60 mg kg^-1^) than that of ‘Nipponbare’ (37.3 mg kg^-1^). Among the six recombinant homozygous lines, two from nos. 29 and 475 exhibited similar Zn concentrations to those of the wild homozygous lines, whereas two from nos. 734 and 757 showed significantly lower values (52.5 and 50.2 mg kg^-1^, respectively) than those of the wild homozygous lines (70.4 and 70.9 mg kg^-1^, respectively). These strongly indicate that the genomic region between RM24085 and RM24165 is not responsible for grain Zn concentration. In addition, the concentration of nos. 734 and 757 recombinant homozygous lines were intermediate between that of ‘Nipponbare’ (37.3 mg kg^-1^) and W1627 (61.4 mg kg^-1^), suggesting that two W1627 factors associated with partial upregulation of grain Zn concentration may exist in RM24165-RM24218 and RM24244-RM566 regions. These regions were further examined with other two recombinant homozygous lines from nos. 249 and 288. The Zn concentration of no. 249 recombinant homozygous line was also an intermediate value of 49.7 mg kg^-1^, confirming that replacement of ‘Nipponbare’ genomic region between RM24244 and RM566 caused partial reduction of Zn concentration. The recombinant homozygous line from no. 288 showed a considerably low value (41.9 mg kg^-1^) similar to that of ‘Nipponbare’ (37.3 mg kg^-1^), indicating that the genomic region between RM24085 and RM24211 is not related to the regulation of grain Zn concentration. Taken together with the result of no. 734 recombinant homozygous line, our findings indicate that the other W1627 factor may be located in the genomic region between RM24211 and RM24218.

The above results indicate that the *qGZn9* putative region contains two independent loci for grain Zn concentration. They are named *qGZn9a* and *qGZn9b*: *qGZn9a* was estimated to be in a 190 kb region between RM24211 and RM24218, and *qGZn9b* was estimated to be in an approximately 950 kb region between RM24244 and RM566 (Fig 3). The genetic dissection of the *qGZn9* region indicated that the high grain Zn concentration observed in MN91 is additively controlled by the two linked loci, *qGZn9a* and *qGZn9b*.

### Candidate genes for *qGZn9a*

We reached genes in the candidate regions of *qGZn9a* and *qGZn9b*. Although more than 100 genes are located in the candidate region of *qGZn9b*, only eight genes are predicted in the *qGZn9a* candidate region according to RAP-DB (Table 2) [26]. Among these, the coding regions of the three genes (i.e., Os09g0383000, Os09g0384601, and Os09g0384900) were not amplified by PCR using several combinations of primers.

**Table 2:** List of the candidate genes for *qGZn9a*.

Gene locus ID ^1)^	Description ^1)^	Presence or absence ^2)^	
		*O*. *sativa* ‘Nipponbare’	*O*. *meridionalis* W1627
Os09g0382300	Cyclin-D2-1	+	+
Os09g0382400	Conserved hypothetical protein	+	+
Os09g0382500	Conserved hypothetical protein	+	+
Os09g0383000	Plant disease resistance response protein domain containing protein	+	−
Os09g0383300	Hypothetical conserved gene	+	+
Os09g0383400	DEAD-like helicase, N-terminal domain containing protein	+	+
Os09g0384601	Hypothetical gene	+	−
Os09g0384900	Protein of unknown function DUF295 family protein	+	−

^1)^ Gene locus ID and description are based on the Rice Annotation Project Database.

^2)^ Presence and absence of the gene are shown as “+” and “-”, respectively, estimated by the PCR and sequencing experiments.

We also analysed next-generation sequencing data of the introgression line having the W1627 chromosomal segment covering both *qGZn9a* and *qGZn9b* loci, however, no homologous sequences of the three genes were detected (S2 Fig). These regions may be deleted or transferred to other chromosomal regions in *O*. *meridionalis*. Although we do not know the gene responsible for *qGZn9a*, one of the three missing or additional genes at the *qGZn9a* region in *O*. *meridionalis* W1627 might cause a drastic change in grain Zn concentration. In addition, we also found that most of the lines having W1627 chromosomal segments at *qGZn9a* showed low fertility rates (< 50%, S3 Fig). We, however, do not know that the reduction of fertility was caused by the same gene controlling grain Zn concentration or a different gene linked to the *qGZn9* candidate region.

## Discussion

### *O*. *meridionalis* is a useful genetic resource for biofortification of grain Zn levels

In this study, we compared grain Zn concentrations of A-genome wild rice species that have cross-compatibility with those of cultivated rice *O*. *sativa*. Among the four wild rice accessions, *O*. *meridionalis* W1627 showed the highest grain Zn concentration (Fig 1C). This characteristic is widely observed in this species (Fig 1D). *O*. *meridionalis* is naturally found in swampy areas in Australia, completely isolated from cultivated rice *O*. *sativa* [27]. According to the phylogenetic analyses of A-genome species, *O*. *meridionalis* is the most distantly-related wild rice to *O*. *sativa* [28,29]. Some studies have reported that *O*. *meridionalis* shows heat tolerance that is associated with Rubisco activity [30–32], but their application in rice breeding has not been achieved.

As found in this study, wild relatives of rice have the potential to increase grain Zn concentrations for the biofortification of cultivated rice *O*. *sativa*. Previously, grain Zn concentrations of cultivated rice, *O*. *sativa*, and three wild rice species, *O*. *rufipogon*, *O*. *officinalis*, and *O*. *granulata*, were compared, and *O*. *officinalis* was found to contain a high grain Zn concentration [33]. Since *O*. *officinalis* has the CC genome constitution, it is quite difficult to transfer wild useful traits to cultivars with the AA genome. Compared to non-A-genome wild relatives, *O*. *meridionalis* has cross-compatibility with *O*. *sativa*. Therefore, we produced BRILs between *O*. *meridionalis* W1627 and *O*. *sativa* Japonica ‘Nipponbare’ to investigate genetic control of high grain Zn concentration. Genotyping analysis of the BRILs showed that the average wild introgression ratio was 4.5%, which is lower than the theoretical value of 12.5%. Some chromosomal segments of *O*. *meridionalis* W1627 were not present in any of BRILs (Fig 2A). Genes in these segments may cause sterility between *O*. *sativa* and *O*. *meridionalis* [22], since they are relatively differentiated from each other. Nevertheless, our BRILs were found to be useful for detecting loci controlling grain Zn concentration and are applicable for identifying genes controlling useful agronomic traits in *O*. *meridionalis*.

### QTLs for grain Zn concentration detected between *O*. *meridionalis* and *O*. *sativa*

We evaluated grain Zn concentration of the 151 BRILs and ‘Nipponbare’ in the paddy field. *O*. *meridionalis* does not produce seeds under natural day-length conditions in Japan. Several lines showed higher grain Zn levels than those of ‘Nipponbare’ (Fig 2C). Based on the QTL analysis, we detected four loci for grain Zn concentration (Table 1). At all the loci, *O*. *meridionalis* W1627 alleles had increasing effects on grain Zn concentration (Table 1). Several studies have been conducted to detect the loci involved in the control of grain Zn concentration in rice [2], but no responsible genes related to the QTL have been isolated. Using Asian wild rice materials, three QTLs were detected in the control of grain Zn concentration between *O*. *sativa* Indica ‘Teqing’ and an accession of *O*. *rufipogon* in Yunnan, China [34]. One of the QTLs on chromosome 8 was repeatedly detected in experiments conducted over two years. The *O*. *rufipogon* allele of the accession at the QTL was found to increase grain Zn concentration with the largest phenotypic variation (11–19%). To our knowledge, no QTL analysis has been conducted thus far to detect the loci for grain Zn concentration using *O*. *meridionalis*. Previously, a segregating population between two rice cultivars, ‘Teqing’ and ‘Lemont’, was used to detect QTLs for grain Zn concentration. One QTL on chromosome 9, *qZn9*, was detected close to RM3909 [35], which is relatively far (4.6 Mb) from the *qGZn9* candidate region in this study. Since the QTL detected in our study is a trait associated with *O*. *meridionalis*, therefore, *qGZn9* may be different from the region detected in previous studies.

A genetic dissection study showed that the *qGZn9* candidate region contained two linked loci, *qGZn9a* and *qGZn9b* (Fig 3). Several QTLs have been found to consist of two linked loci, based on extensive genetic analysis. For example, *Hd3a*, an important flowering QTL encoding the rice florigen gene, was identified through genetic dissection at the *Hd3* locus [36]. *Gn1a*, which encodes a cytokine oxidase that controls grain number in cultivated rice, was identified through the genetic dissection at *Gn1* [37]. Further genetic analyses on *qGZn9a* and *qGZn9b* will help to identify the causal genes at both loci.

### Genes underlying *qGZn9a*

In the mapping experiment on *qGZn9a*, we narrowed down the candidate region to an approximately 190 kb genomic segment carrying eight putative genes based on the ‘Nipponbare’ genome sequence database. Of these, three genes were found to be missing in the corresponding region of *O*. *meridionalis* W1627, based on PCR and next-generation sequencing analysis (Table 2 and S2 Fig). No annotated genes were involved in Zn transport or homeostasis among the eight genes. Interestingly, in the progeny test, we found that most of lines carrying the W1627 chromosomal segment covering *qGZn9a* showed extremely low fertility rates (S3 Fig). In contrast, progeny carrying the W1627 chromosomal segment covering only the *qGZn9b* region showed higher fertility. Since low fertility is negatively correlated with grain Zn concentration, accumulation of Zn may be enhanced in the small number of fertile seeds. Interestingly, Os09g0384900, one of the three missing genes in the *qGZn9a* candidate region of *O*. *meridionalis* W1627, is highly expressed in the developing anther (1.2–1.5 mm), according to the rice expression database RiceXpro (http://ricexpro.dna.affrc.go.jp/). Although the function of the protein encoded by Os09g0384900 in pollen development is not clearly known, it is a strong candidate gene for *qGZn9a*.

In addition, the inheritance of the *qGZn9* effect on grain Zn concentration is sporophytically controlled, as grain Zn levels of the introgression lines with heterozygous chromosome constitution at *qGZn9* are similar to those of ‘Nipponbare’. This finding suggests that the difference between the grain Zn concentrations of *O*. *sativa* and *O*. *meridionalis* might be regulated before Zn is transferred to the seeds. In previous studies, the node has been considered an important tissue that controls mineral distribution [38,39]. The gene encoded at *qGZn9b* might play roles in the node.

### Utilization of *qGZn9* for biofortification of rice

We found that the *O*. *meridionalis* allele at *qGZn9a* increases grain Zn concentration, but causes low seed fertility (S3 Fig). Although a detailed evaluation of the effect of *qGZn9a* on grain Zn concentration is required, the reduction in fertility is not conducive to rice breeding. In contrast, lines having the W1627 chromosomal segment covering only the *qGZn9b* region had lower fertility reduction than those covering the *qGZn9a* region did (S3 Fig). Therefore, the *O*. *meridionalis* allele at *qGZn9b* can be used for biofortification of Zn in cultivated rice, *O*. *sativa*. Generations of near isogeneic lines for *qGZn9a* and *qGZn9b* are required to evaluate the independent effects of the two loci on grain Zn concentration. Identification of the causal genes for *qGZn9a* and *qGZn9b* will shed light on how Zn is distributed to the seeds. Once DNA markers detecting the causal mutation at *qGZn9b* or those tightly linked to it have been developed, they will be useful for marker-assisted selection of lines with high grain Zn levels. A sustainable strategy to alleviate Zn deficiency in many developing countries can be achieved when these lines are successfully developed. In addition, the development of new cultivars using favourable traits from *O*. *meridionalis* would be advantageous for the conservation of wild rice, as their suitable growth habitats are shrinking.

## Supporting information

S1 Fig SSR markers used for QTL analysis and positions of the QTLs responsible for grain Zn concentration in *O*. *sativa* ‘Nipponbare’ and *O*. *meridionalis* W1627. Dotted lines beside chromosomes 1 and 9 show chromosomal segments of W1627 introgressed in MN91, one of the BRILs used for further genetic analysis.
(PDF)

S2 Fig Sequencing analysis of *qGZn9a* candidate genes. Sequence reads obtained from the introgression line carrying W1627 chromosomal segment covering *qGZn9a* were aligned to the ‘Nipponbare’ genome using the Galaxy software (https://usegalaxy.org/). No sequence reads corresponding to Os09g0383000, Os09g0384601, or Os09g0384900 were obtained.
(PDF)

S3 Fig Fertility of the plants in the progeny test of *qGZn9* linkage analysis. Two types of homozygous plants with wild (W) and recombinant (R) chromosomes were generated from six recombinant lines. White and black boxes indicate ‘Nipponbare’ and W1627 chromosomal segments, respectively.
(PDF)

S1 Table Wild rice accessions used in this study. (PDF)

S2 Table Grain size and weight for parental lines (*Oryza sativa* ‘Nipponbare’ and *O*. *meridionalis* W1627) and MN91, a BRIL used for genetic analysis of grain Zn concentration. Data are presented as mean ± s.d. (n = 3).
(PDF)

S3 Table Comparison of seed producing parameters between *Oryza sativa* ‘Nipponbare’ and MN91 grown in the paddy field. Data are presented as mean ± s.d. (n = 6).
(PDF)
