# Supplementary material for: Detection of quantitative trait loci controlling grain zinc concentration using Australian wild rice, Oryza meridionalis, a potential genetic resource for biofortification of rice
Source: PLoS One. 2017 Oct 27;12(10):e0187224. doi: 10.1371/journal.pone.0187224 (PMC5659790; doi:10.1371/journal.pone.0187224)
Supplement: S1 Fig — Dotted lines beside chromosomes 1 and 9 show chromosomal segments of W1627 introgressed in MN91, one of the BRILs used for further genetic analysis. (PDF) [file pone.0187224.s001.pdf]

Ishikawa et al. S1 Fig.

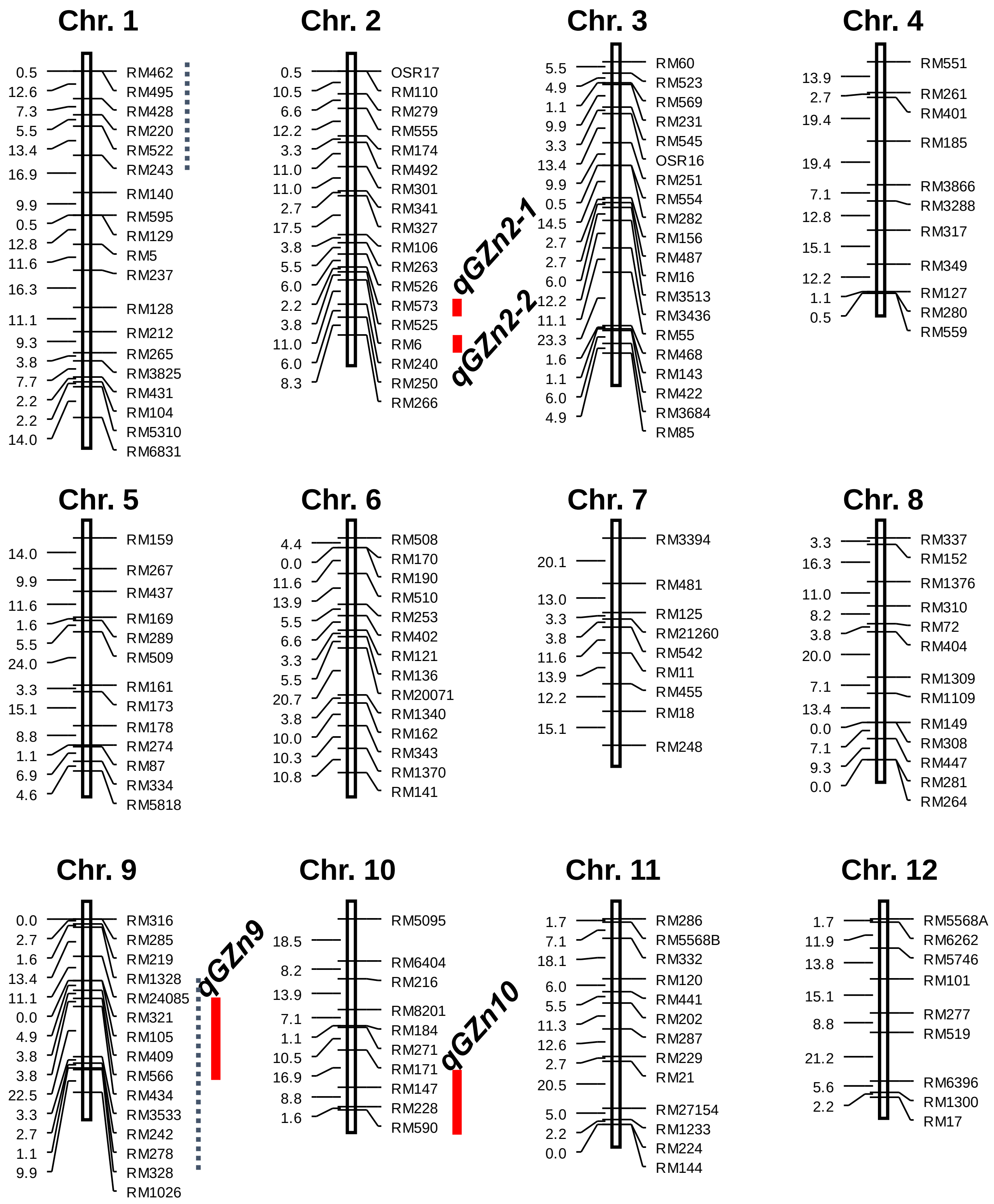

S1 Fig. SSR markers used for QTL analysis and positions of the QTLs responsible for grain Zn concentration in *O. sativa* 'Nipponbare' and *O. meridionalis* W1627. Dotted lines beside chromosomes 1 and 9 show chromosomal segments of W1627 introgressed in MN91, one of the BRILs used for further genetic analysis.
