# Supplementary material for: Detection of quantitative trait loci controlling grain zinc concentration using Australian wild rice, Oryza meridionalis, a potential genetic resource for biofortification of rice
Source: PLoS One. 2017 Oct 27;12(10):e0187224. doi: 10.1371/journal.pone.0187224 (PMC5659790; doi:10.1371/journal.pone.0187224)
Supplement: S2 Fig — Sequence reads obtained from the introgression line carrying W1627 chromosomal segment covering qGZn9a were aligned to the ‘Nipponbare’ genome using the Galaxy software (https://usegalaxy.org/). No sequence reads corresponding to Os09g0383000, Os09g0384601, or Os09g0384900 were obtained. (PDF) [file pone.0187224.s002.pdf]

Ishikawa et al. S2 Fig.

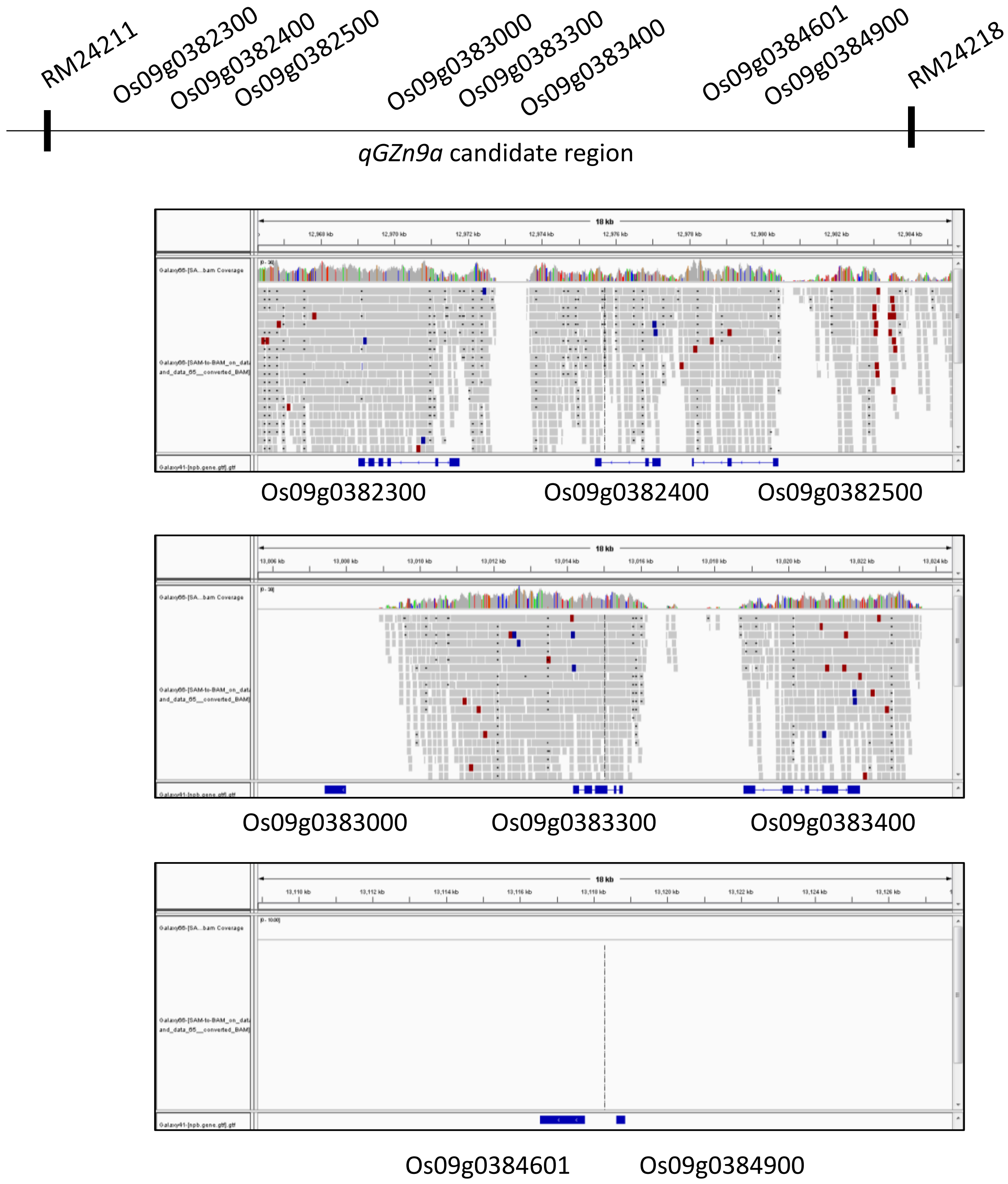

S2 Fig. Sequencing analysis of *qGZn9a* candidate genes. Sequence reads obtained from the introgression line carrying W1627 chromosomal segment covering *qGZn9a* were aligned to the 'Nipponbare' genome using the Galaxy software (https://usegalaxy.org/). No sequence reads corresponding to Os09g0383000, Os09g0384601, or Os09g0384900 were obtained.
