# Supplementary material for: Detection of quantitative trait loci controlling grain zinc concentration using Australian wild rice, Oryza meridionalis, a potential genetic resource for biofortification of rice
Source: PLoS One. 2017 Oct 27;12(10):e0187224. doi: 10.1371/journal.pone.0187224 (PMC5659790; doi:10.1371/journal.pone.0187224)
Supplement: S3 Fig — Two types of homozygous plants with wild (W) and recombinant (R) chromosomes were generated from six recombinant lines. White and black boxes indicate ‘Nipponbare’ and W1627 chromosomal segments, respectively. (PDF) [file pone.0187224.s003.pdf]

Ishikawa et al. S3 Fig.

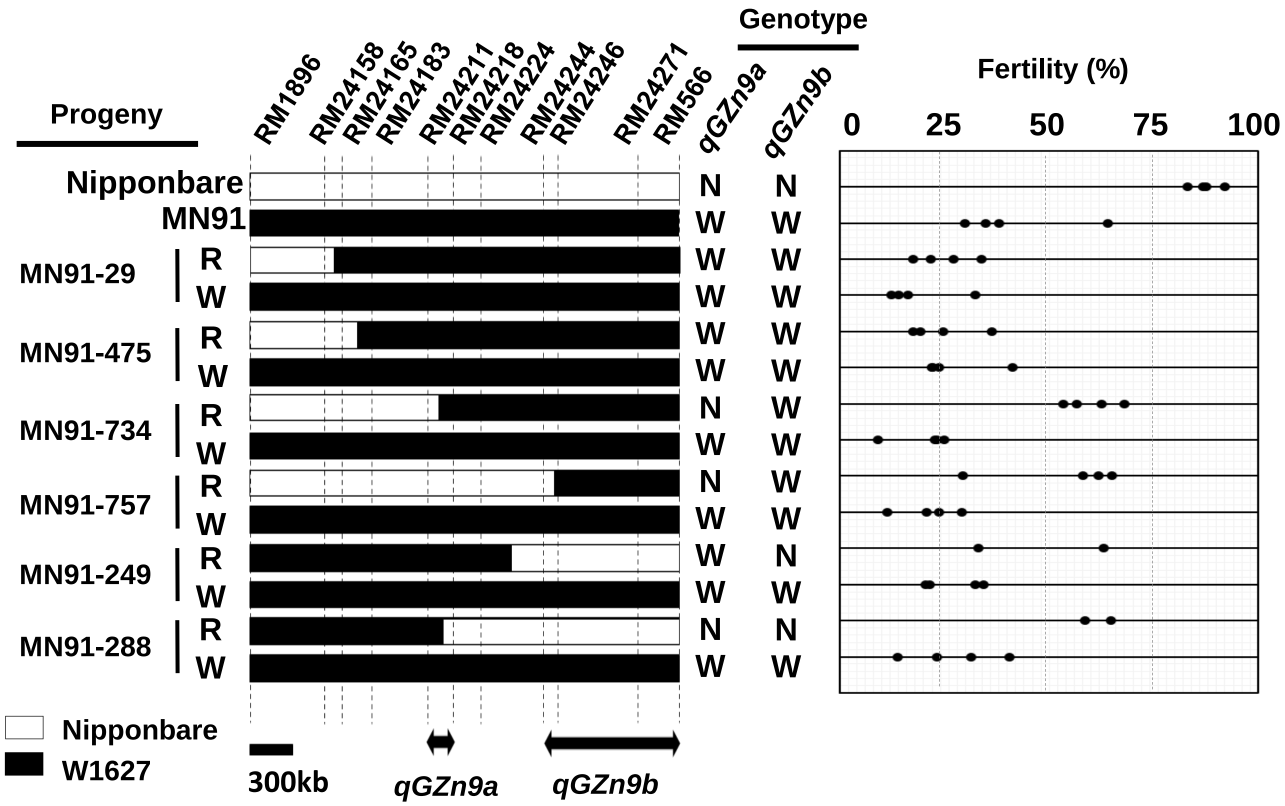

S3 Fig. Fertility of the plants in the progeny test of *qGZn9* linkage analysis. Two types of homozygous plants with wild (W) and recombinant (R) chromosomes were generated from six recombinant lines. White and black boxes indicate 'Nipponbare' and W1627 chromosomal segments, respectively.
