# Supplementary material for: Detection of quantitative trait loci controlling grain zinc concentration using Australian wild rice, Oryza meridionalis, a potential genetic resource for biofortification of rice
Source: PLoS One. 2017 Oct 27;12(10):e0187224. doi: 10.1371/journal.pone.0187224 (PMC5659790; doi:10.1371/journal.pone.0187224)
Supplement: S1 Table — (PDF) [file pone.0187224.s004.pdf]

S1 Table. Wild rice accessions used in this study.

Species	Accession no. [1)]	Country of origin
O. rufipogon	W630	Myanmar
O. glumaepatula	W1169	Cuba
O. barthii	W1152	Unknown (Africa)
O. meridionalis	W1627	Australia
"	W1298	"
"	W1561	"
"	W1629	"
"	W1631	"
"	W1635	"
"	W2069	"
"	W2080	"
"	W2081	"
"	W2103	"
"	W2116	"

[1)] Accession code is after National Institute of Genetics, Mishima, Japan
