# Supplementary material for: Detection of quantitative trait loci controlling grain zinc concentration using Australian wild rice, Oryza meridionalis, a potential genetic resource for biofortification of rice
Source: PLoS One. 2017 Oct 27;12(10):e0187224. doi: 10.1371/journal.pone.0187224 (PMC5659790; doi:10.1371/journal.pone.0187224)
Supplement: S2 Table — Data are presented as mean ± s.d. (n = 3). (PDF) [file pone.0187224.s005.pdf]

S2 Table. Grain size and weight for parental lines (*Oryza sativa* ‘Nipponbare’ and *O. meridionalis* W1627) and MN91, a BRIL used for genetic analysis of grain Zn concentration. Data are presented as mean ± s.d. (n=3).

Grain trait	‘Nipponbare’	W1627	MN91
Length (mm)	4.77 ± 0.05	5.20 ± 0.09	4.58 ± 0.02
Width (mm)	2.72 ± 0.08	1.72 ± 0.03	2.68 ± 0.03
Thickness (mm)	1.96 ± 0.06	1.21 ± 0.04	1.95 ± 0.03
Weight (mg)	20.56 ± 0.70	8.46 ± 0.30	18.87 ± 0.08
