# Supplementary material for: Detection of quantitative trait loci controlling grain zinc concentration using Australian wild rice, Oryza meridionalis, a potential genetic resource for biofortification of rice
Source: PLoS One. 2017 Oct 27;12(10):e0187224. doi: 10.1371/journal.pone.0187224 (PMC5659790; doi:10.1371/journal.pone.0187224)
Supplement: S3 Table — Data are presented as mean ± s.d. (n = 6). (PDF) [file pone.0187224.s006.pdf]

S3 Table. Comparison of seed producing parameters between *Oryza sativa* ‘Nipponbare’ and MN91 grown in the paddy field. Data are presented as mean ± s.d. (n=6).

Seed producing parameter	‘Nipponbare’	MN91
No. of panicles	13.5 ± 1.5	15.0 ± 1.8
No. of spikelets per panicle[1)]	112.6 ± 7.7	132.8 ± 10.1
Fertility (%)	91.6 ± 2.7	75.5 ± 3.5

[1)] No. of spikelets per panicle for each plant was estimated by the average of five panicles
